# Supplementary material for: Can the Timed and Targeted Counseling Model Improve the Quality of Maternal and Newborn Health Care? A Process Analysis in the Rural Hoima District in Uganda
Source: Int J Environ Res Public Health. 2021 Apr 21;18(9):4410. doi: 10.3390/ijerph18094410 (PMC8122283; doi:10.3390/ijerph18094410)
Supplement: Supplementary file 1 [file ijerph-18-04410-s001.zip › ijerph-1114913-supplementary.pdf]

International Journal of
Environmental Research and Public Health

MDPI

Supplementary Table S1. Characteristics of the pregnant women (N=616) and VHTs (N=64) included in the study.

Sub-County	Number of Villages	Number of VHTs by Gender	Number of Included Households
Bugembe	10	8 (M:4, F:4)	99
Buseruka	7	8(M:3, F:5)	75
Busisi	7	7 (M:2, F:5)	69
Kigorobya	7	8(M:5, F:3)	81
Kitoba	8	9(M:3, F:6)	78
Lower Kabwoyo	8	8 (M:6, F:2)	79
Mparo	8	8 (M:2, F:6)	56
Lower Kabwoyo	9	9 (M:5, F:4)	79
Total	64	65 (M:30, F:35)	616

Note: VHTs = Village Health Team., M = male, F = female.

Supplementary Table S2. Quality of care during pregnancy and childbirth.

Service or Practice	%	
	**Baseline**	**Implementation**
Quality of ANC services		
Quality of ANC service		18.5%
Contributing Services to Quality of ANC		
Woman given folic acid during ANC	28.8%	95.8%
Woman given Fansidar (IPT) during ANC	78.6%	77.9%
Woman tested for HIV and results given	91.0%	76.0%
Woman given deworming during Pregnancy	n.a.	69.1%
Woman vaccinated for TT once	n.a.	56.4%
ANC First Trimester	24.4%	53.9%
4th ANC or more	55.1%	52.2%
Woman tested for syphilis	n.a.	41.9%
Quality of ANC care at home		
Quality of ANC care at home		21.6%
Contributing practices to ANC care at home		
Woman having adequate rest	n.a.	81.5%
Woman having extra meal	n.a.	78.4%
Woman sleeping LLTIN	74.0%	49.3%
Hand washing facility at the home	n.a.	41.8%
Appropriate childbirth practices		
Appropriate Childbirth Practices		28.7%
Contributing practices to childbirth		
Woman plans to deliver at Health Facility	n.a.	76.5%
Woman discussed a birth plan	13.2%	76.0%
Delivery at the Health Facility	80.0%	90.8%
Woman has a clean birthing kit	89.9%	49.9%
Quality of Newborn care at home		
Newborn care at home		29.2%
Contributing practices to newborn care		
Mother visited during the 1st week	n.a.	89.6%
Baby sleeping under an LLTIN	74.9%	86.8%
Baby practicing exclusive breastfeeding	60.9%	85.5%
Baby suckling well	n.a.	79.4%
Birth weight record	n.a.	63.6%
Vaccination	n.a.	48.2%

Note: ANC: Antenatal care; LLITN: Long-lasting insecticide-treated mosquito net; IPT: Intermittent presumptive treatment of malaria; TT: Tetanus Toxoid vaccination; n.a.: Not assessed.

Supplementary Table S3. Services and practices during pregnancy.

Variables	ANC 1 (596)		ANC 2 (528)		ANC 3 (426)		ANC 4 or more (311)	
	N	%	N	%	N	%	N	%
Goal-oriented ANC services								
TT vaccination for the mother	292	49.0%	216	40.9%			NA	
HIV testing & results received	388	65.1%	294	55.7%	207	48.6%	14	4.5%
Woman given IPT (Fansidar)	297	49.8%	332	62.9%	234	54.9%	26	8.4%
Woman given folic acid	501	84.1%	458	86.7%	373	87.6%	282	90.7%
Woman given dewormers	NA		360	68.2%	265	62.2%	NA	
Woman tested for syphilis	NA		225	42.6%	143	33.6%	NA	
Practices at Home during Pregnancy								
Woman sleeping under LLITN	150	25.2%	174	33.0%	18	4.2%	0	0.0%
Woman having adequate rest	108	18.1%	144	27.3%	169	39.7%	282	90.7%
Woman eating an extra meal	109	18.3%	136	25.8%	161	37.8%	274	88.1%
Handwashing facility available	55	9.2%	67	12.7%	86	20.2%	160	51.4%

Note: LLITN: Long-lasting insecticide-treated mosquito net; IPT: Intermittent presumptive treatment of malaria; TT: Tetanus Toxoid vaccination; ANC: Antenatal care; NA: Not assessed.

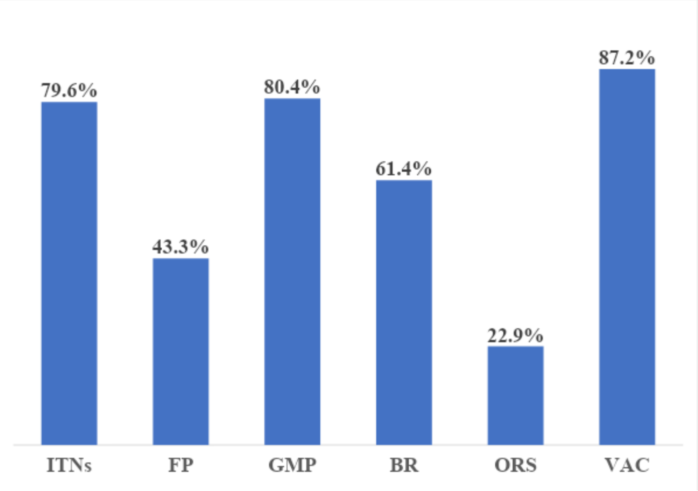

Supplementary Figure S1. Newborn care at 6 weeks after childbirth. ITNs: Baby sleeping under an insecticide-treated mosquito net; FP: Mother prepared to use family planning method; GMP: Child Health Card plotted at immunization; BR: Baby and mother practicing exclusive breastfeeding; ORS: Mother taught how to prepare ORS for diarrhea care; VAC: Baby vaccinated for Polio 1, and DPT.

Supplementary Table S4. Results of a Chi-Square test on the 5 outcome variables.

Indicator	Number and Percentage		*p*-value
	Baseline	Outcome	
First ANC in first trimester	168(24.3%)	321(53.9%)	<0.001
Quality of ENC	1(0.5%)	159(29.2%)	
Quality of ANC	3(0.4%)	111(17.9%)	
Exclusive breastfeeding	125(60.0%)	465(85.5%)	
4th or more ANC	381(55.1%)	311(50.5%)	0.153

Notes: ANC; Antenatal care, ENC: Essential newborn care.
